# Notes on the Reproductive Ecology and Description of the Preimaginal Morphology of *Elaphrus sugai* Nakane, the Most Endangered Species of *Elaphrus* Fabricius (Coleoptera: Carabidae) Ground Beetle Worldwide

**DOI:** 10.1371/journal.pone.0159164

**Published:** 2016-07-14

**Authors:** Kôji Sasakawa

**Affiliations:** 1 Laboratory of Zoology, Department of Science Education, Faculty of Education, Chiba University, Yayoi-cho, Inage-ku, Chiba, Japan; 2 Department of General Systems Studies, Graduate School of Arts and Sciences, University of Tokyo, Komaba, Meguro-ku, Tokyo, Japan; CNRS, FRANCE

## Abstract

Elucidating the basic life-history of endangered species is the first important step in the conservation of such species. This study examined the reproductive ecology and the preimaginal morphology of the endangered ground beetle *Elaphrus sugai* Nakane (Coleoptera: Carabidae); currently, the Watarase wetland of the central Kanto Plain, Japan is the only confirmed locality of this beetle species. Laboratory rearing of reproductive adults collected in early April revealed that females can lay more than 131 eggs. Eggs were laid in mud, without an egg chamber. Larvae reached adulthood when fed a diet of mealworms, indicating that *E*. *sugai* larvae are insect larvae feeders. An earthworm diet, the optimal diet for larvae of a congeneric species (*E*. *punctatus* Motschulsky), was lethal to *E*. *sugai* larvae. The egg stage was 3–4 days in duration under a 16L8D cycle (22°C). The duration from hatching to adult eclosion was 23–42 days at various temperatures simulating those of the reproductive period. Larval morphology was similar to that of consubgeneric species described previously. The pupa is unusual, in that the setae on the abdominal tergites are long (twice as long as those of the abdominal segment) and have somewhat “coiled” apices. Finally, the current endangered status of *E*. *sugai* was compared to that of *E*. *viridis* Horn, which has been regarded as the most endangered species of the genus worldwide.

## Introduction

Environmental degradation caused by human activities and the resulting increases in extinction risks are problematic worldwide, including Japan. The current (2012–2013) Red List prepared by the Japanese Ministry of the Environment lists 3,597 species as threatened (i.e., “Critically Endangered”, “Endangered”, and “Vulnerable”), compared with the 3,155 species of the 2007 list, reflecting addition of over 400 species [1,2]. The current number of threatened insect species is 1.50-fold higher than that in the 2007 list (an increase from 239 to 358 species). This rate of increase is highest in ten taxa examined, namely mammals, birds, reptiles, amphibians, brackish-water/freshwater fishes, insects, shellfish, other invertebrates (arachnids, crustaceans, etc.), plants I (vascular plants), and plants II (nonvascular plants). Among the insect groups, ground beetles (Coleoptera: Carabidae) and moths (Lepidoptera) that inhabit wetlands or grasslands and water beetles (Coleoptera: Dytiscidae, Gyrinidae) that inhabit paddy fields or storage ponds are severely threatened [1]. Elucidating basic information such as the precise range and life history is the first essential step in conservation.

*Elaphrus sugai* Nakane is a small carabid (about 8 mm in body length) that inhabits wetlands and is listed as “Endangered” in the current Red List [3]. The species has been recorded from two wetlands located in the central Kanto Plain, but there have been no records since 1952 from one of these two localities (the Sugao pond, Ibaraki Prefecture) [4]. Currently, the type locality, the Watarase wetland (Tochigi Prefecture) is the only confirmed locality. The reeds of this wetland have been burned annually for approximately the past 60 years, which has contributed to maintenance of the *E*. *sugai* habitat [5]. However, after the Great East Japan Earthquake of March 11, 2011, burning was cancelled the following 2 years. This, together with recent drying of the wetland environment, may compromise the *E*. *sugai* habitat. Indeed, collection of this beetle has not been reported since 2011, despite intensive field surveys by the Government in 2012–2014 [6,7,8].

This study describes the reproductive ecology and preimaginal morphology of *E*. *sugai*, obtained from laboratory rearing using reproductive adults collected in 2013 and 2014. To my knowledge, this was the first formal record of the species since 2011. As the ecology and preimaginal morphology of the species have not been described previously, the results presented here will aid future conservation efforts.

## Materials and Methods

**Fig 1:**
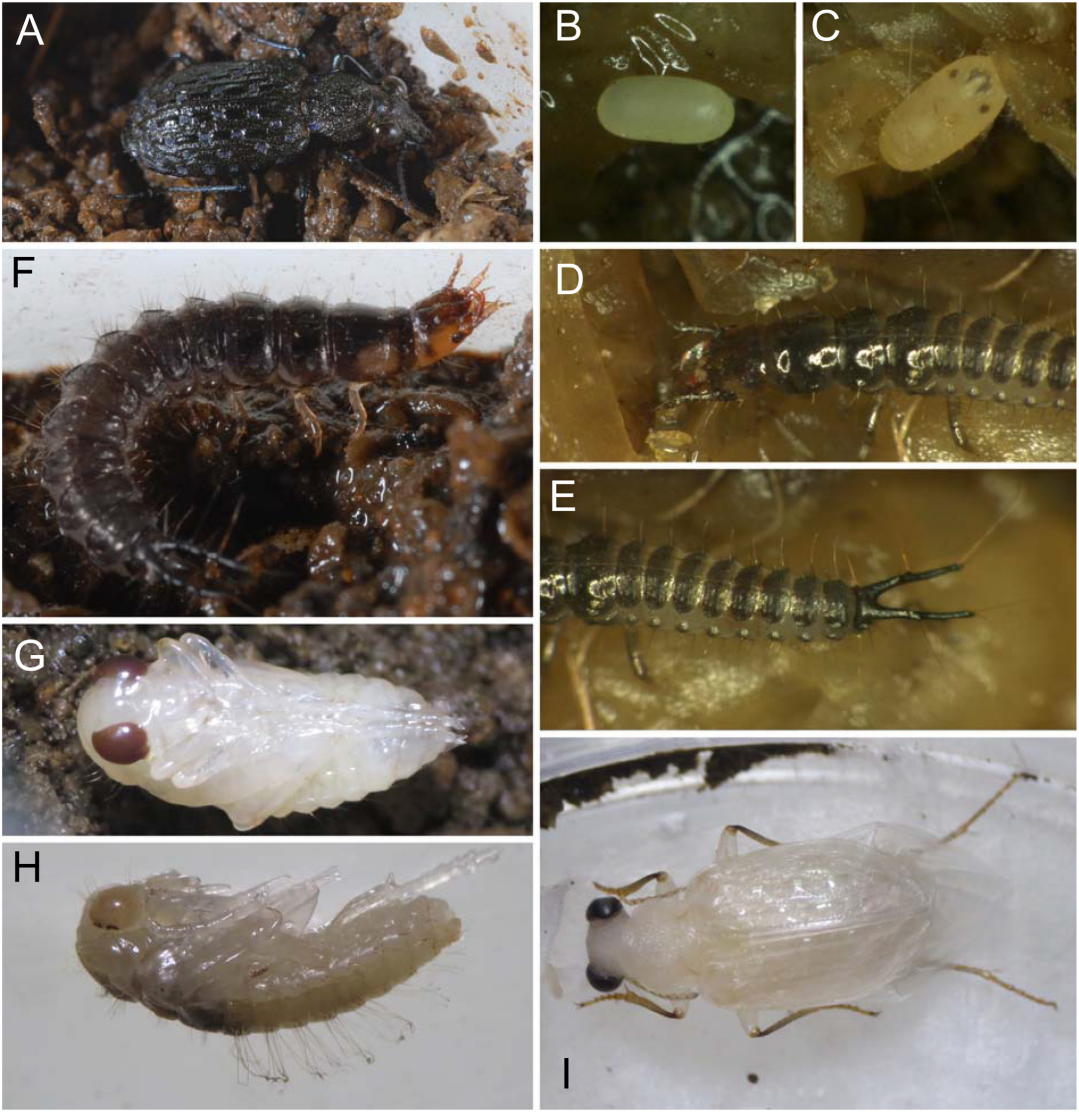
Life history of *E*. *sugai*. (A) Reproductive female in 2013, (B) an egg laid within 24 h, (C) a 2-days old egg, (D) the anterior half of a first-instar larva, (E) the posterior half of the same first instar individual, (F) a third-instar larva, (G) a pupa, (H) another pupa from lateral view, to show setae on the dorsal side, (I) a newly-emerged adult. The photos vary in magnification.

Laboratory rearing was performed using reproductive adults that were collected at the Watarase wetland on May 7–8, 2013 (one female) and April 12–13, 2014 (two females and three males) and immediately transported to a laboratory at the University of Tokyo (Komaba, Meguro-ku, Tokyo). No specific permissions were required for collecting this beetle species at this locality (see also Discussion). The beetles were stored at 5°C until the experiments commenced. As the ecology of the species was completely unknown, both adults and larvae were reared under various conditions (e.g., different cages, diets, temperatures, and photoperiods). The details are shown in Table 1. In both adults and larvae, insects were reared individually except adult rearing in 2014, in which a randomly-chosen male was added to each female arena for 24 h every 6–8 days and they were allowed to mate. Although some individuals were killed at the larval or pupal stages to obtain specimens for description, most larvae reared to adulthood if possible (Fig 1). For reproductive ecology, (i) female ovipositioning behavior and fecundity, (ii) larval feeding habits, and (iii) the developmental durations of the preimaginal stages, were examined. The fecundities were calculated based on two females that were thought to exhibit normal egg production. Data on larval feeding habits were obtained from choice and no-choice dietary experiments performed in 2014. Developmental durations were calculated based on individuals for which hatching or molting was confirmed (for individuals that hatched or molted in the soil, hatching and molting dates could not be determined).

**Table 1:** Condition of laboratory rearing of adult and larval *E*. *sugai*.

	Year	
	2013	2014
Starting date	9 May	16 April
Photoperiod and temperature	16L8D (22°C)	16 April–13 May: 16L(20°C)8D(15°C); 13 May–12 July: 16L(22°C)8D(17°C); 12 July–: 16L8D(24°C). Some individuals are reared on room temperature (approximately 20°C) with natural photoperiod.
Rearing instruments for adults^1^^,^ ^2^	Plastic box (10.0×10.0×3.0 cm) in which a Petri dish (9.0 cm diameter, 1.5 cm high) is placed. The Petri dish is filled with flooded peat at the collection site, and the other part in the box is filled to a depth of 1.5 cm with flooded garden soil. Moistened moss is placed on the soil surface.	Petri dish (9.0 cm diameter, 1.5 cm high) filled to a depth of 0.5 cm with flooded garden soil. Moistened moss is placed on the soil surface.
Rearing instruments for larvae^1^^,^ ^3^^,^ ^4^	Egg, L1, and L2: Petri dishes (3.5 cm diameter, 1.0 cm high) filled to a depth of 0.5 cm with moistened garden soil. L3 and pupa: plastic bottles (6.5 cm diameter, 7.5 cm height) filled to a depth of 4.0 cm with moistened garden soil.	Egg, L1, and L2: Petri dishes (3.5 cm diameter, 1.0 cm high) filled to a depth of 0.5 cm with moistened garden soil. L3 and pupa: plastic bottles (3.5 cm diameter, 3.5 cm height) filled to a depth of 2.0 cm with moistened garden soil.
Adult diet^5^^,^ ^6^	Mealworm (*Tenebrio molitor* larvae)	Mealworm
Larval diet^3^^,^ ^5^^,^ ^6^	Mealworm (for all instars)	Mealworm (for all instars); earthworm (*Pheretima* spp.) (only for L1 as a choice and a non-choice diets); snail (*Bradybaena* spp.) (only for L1 as a choice diet)

^1^Peat and garden soil are frozen at least 24 hours to prevent “biotic contamination”.

^2^The instrument was replaced at 3–4 day intervals to prevent predation by adult on eggs and hatched larvae.

^3^L1, L2, and L3 denote the first, second, and third-larval instars, respectively.

^4^Death and molting are checked daily.

^5^Diet is provided at an *ad libitum* feeding level and replaced on a daily basis.

^6^All diets are cut into piece immediately after being killed.

The preimaginal stages were described based on photographs and specimens of some individuals. Color descriptions are based on color photographs of live individuals at all stages. Morphological descriptions are based on photographs of live eggs, slide-mounted and ethanol-preserved larval specimens (respectively, for detailed morphological observation and measurement), and photographs of live pupae and pupae fixed in absolute ethanol and preserved in 70% ethanol. For slide-mounted larval specimens, five first-instar, two second-instar, and three third-instar individuals were mounted as follows. First, live or freshly-dead individuals were fixed by boiling in water for a few minutes and subsequently preserving in 70% ethanol. Next, the specimens were partly disarticulated, cleaned with 10% potassium hydroxide solution overnight, and dehydrated in absolute ethanol. They were next mounted on glass slides, under cover slips, using Euparal. To prevent deformation by compression, a “frame” of wood fragments or metallic staples was placed between the slide and the cover slip. Other larval specimens were fixed by boiling in water for a few minutes and preserved in 70% ethanol.

The terminology used for most structures follows that of Bousquet & Goulet [9] and Bousquet [10]. Secondary setal counts for the following structures represent counts from both sides combined, unless otherwise stated: the pro-, meso- and meta-notum; abdominal tergites I–VIII; the prosternite; the median sternite; the sternal sclerite; and the pygidium (both dorsal and ventral). The counts of some secondary setae exhibiting marked individual variations (e.g., thoracic nota) were described using the following four categories of Bousquet & Smetana [11]: few (less than 6), moderately numerous (6–15), numerous (16–40), and very numerous (more than 40).

## Results

### Ovipositioning behavior and fecundity

Twenty larvae were obtained from the female in 2013 (Fig 1A). Of these, three were obtained from eggs laid on moistened moss (Fig 1B and 1C), and one from eggs laid on flooded garden soil (i.e., mud). The remaining 16 individuals could not be found at the egg stage, but were found on mud after hatching (Fig 1D and 1E). In 2014, 131 larvae were obtained from one female and 4 from the other female. All larvae were found on mud after hatching. Although the soil in the rearing cages was checked carefully, egg chambers, which have been reported in other carabids [12], were not found.

**Fig 2:**
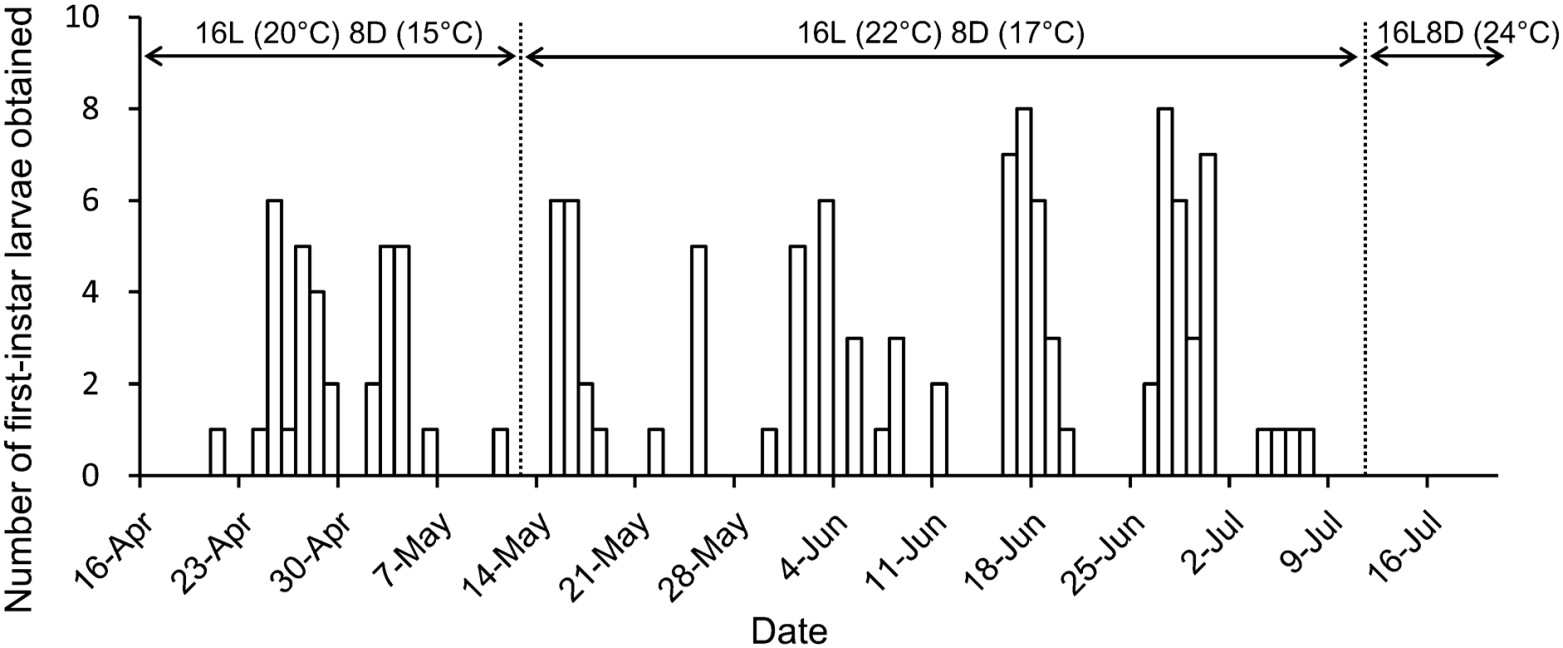
Numbers of first-instar larvae obtained from eggs laid by a female in 2014. Most larvae were obtained within 24 h of hatching.

For the 2013 female, the first larvae hatched on May 15 (6 days after the start of the experiment) from one of two eggs laid on moistened moss on May 12. The second larva hatched on the same day, and subsequent larvae were obtained on May 15 (two individuals), 19 (two), 30 (five), and 31 (four) and June 1 (three), 3 (one), 4 (one) and 5 (two). The fecundity (the number of first-instar larvae obtained per day) was thus 0.95. Of the 131 larvae from the 2014 female, the first was obtained on April 21 (5 days after the start of the experiment). Then, one to eight individuals were obtained daily at 1–5-day intervals until 7 July (Fig 2). The fecundity was thus 1.70.

### Larval feeding habits

Because there was no information on larval feeding habits, all three major types of carabid larval diet (insect larvae, earthworms, and snails/slugs) were provided simultaneously to early hatchlings in 2013. Following Sasakawa [13], commercial mealworms (*Tenebrio molitor* larvae), *Pheretima* earthworms, and *Bradybaena* snails were provided. First-instar larvae ate the mealworms only; thus only mealworms were provided in later work. Most larvae reached subsequent developmental stages, and one became adult (Table 2; note that in 2013, almost half of all larvae were killed [specimens]). In 2014, most larvae were reared on only the mealworm diet and some reached adulthood (Table 3). In both 2013 and 2014, larval mortality was higher in first-instar larvae than in others due to mold on the substrate and/or diet.

**Table 2:** Larvae reared in 2013.

	L1	L2	L3	Pupa	Adult
Number of individuals					
At the initial phase of each developmental stage	20	11	8	3	1 (♀)
Mortalities at each developmental stage	5	1	0	2	–
Killed as specimens	4	2	5	0	–

**Table 3:** Larvae reared in 2014.

	L1	L2	L3	Pupa	Adult
Number of individuals					
At the initial phase of each developmental stage	135	46	22	11	10 (6♂4♀)
Mortalities at each developmental stage	85	24^1^	5	1 (♀)	–
Killed as specimens	4	0	6	0	–

^1^Two individuals on the earthworm diet are included.

The earthworm diet was reexamined in 2014, because successful rearing to adulthood using only an earthworm diet had then been confirmed in a congeneric species, *Elaphrus punctatus* Motschulsky (Sasakawa, unpublished data). Only earthworms were provided to two 1-day old, unfed second-instar *E*. *sugai* that had been reared on a mealworm diet in the first-instar. Under this no-choice condition, the larvae eventually ate some earthworms but died the next day.

### Developmental durations

The following insect individuals were not used when calculating the duration of each developmental stage, because their hatching or molting dates were ambiguous: (i) eggs found apparently after being laid; (ii) first instars found after hatching; and (iii) individuals that molted in the soil. Consequently, few data were available, because most hatching, pupation, and adult eclosion occurred in soil. The number of days [mean (min–max, n)] at the egg stage was 3.3 (3–4, n = 3) at 16L8D (22°C). The durations of the first instar were 4.0 days (3–5 days, n = 2) at 16L8D (22°C), 5 days (n = 1) at 16L (22°C) 8D (17°C), and 5.0 days (4–6 days, n = 4) at 16L (20°C) 8D (15°C). The durations of the second instar were 6.5 days (5–8 days, n = 2) at 16L8D (22°C), 5.4 days (3–10 days, n = 10) at 16L (22°C) 8D (17°C), and 7.0 days (5–8 days, n = 3) at 16L (20°C) 8D (15°C). The durations of the third instar and pupa were 12 days and 4 days (both n = 1), respectively, at 16L8D (22°C).

Although the hatching dates were unclear, the time from first-instar entry to adult eclosion was 26 days for the adult that emerged in 2013. For the 10 adults that emerged in 2014, these times were 16, 19, 21, 23, 24 (three individuals), 25, 28, and 42 days. Of these, the 16-, 19-, and 21-day individuals took only 0–3 days before molting to the second-instar, indicating that these individuals were found several days after hatching. Thus, their data may be unreliable. The time from hatching to adult eclosion was therefore 23–42 days

### Descriptions of the preimaginal stages

Figs 1B–1H and 3–8

**Fig 3:**
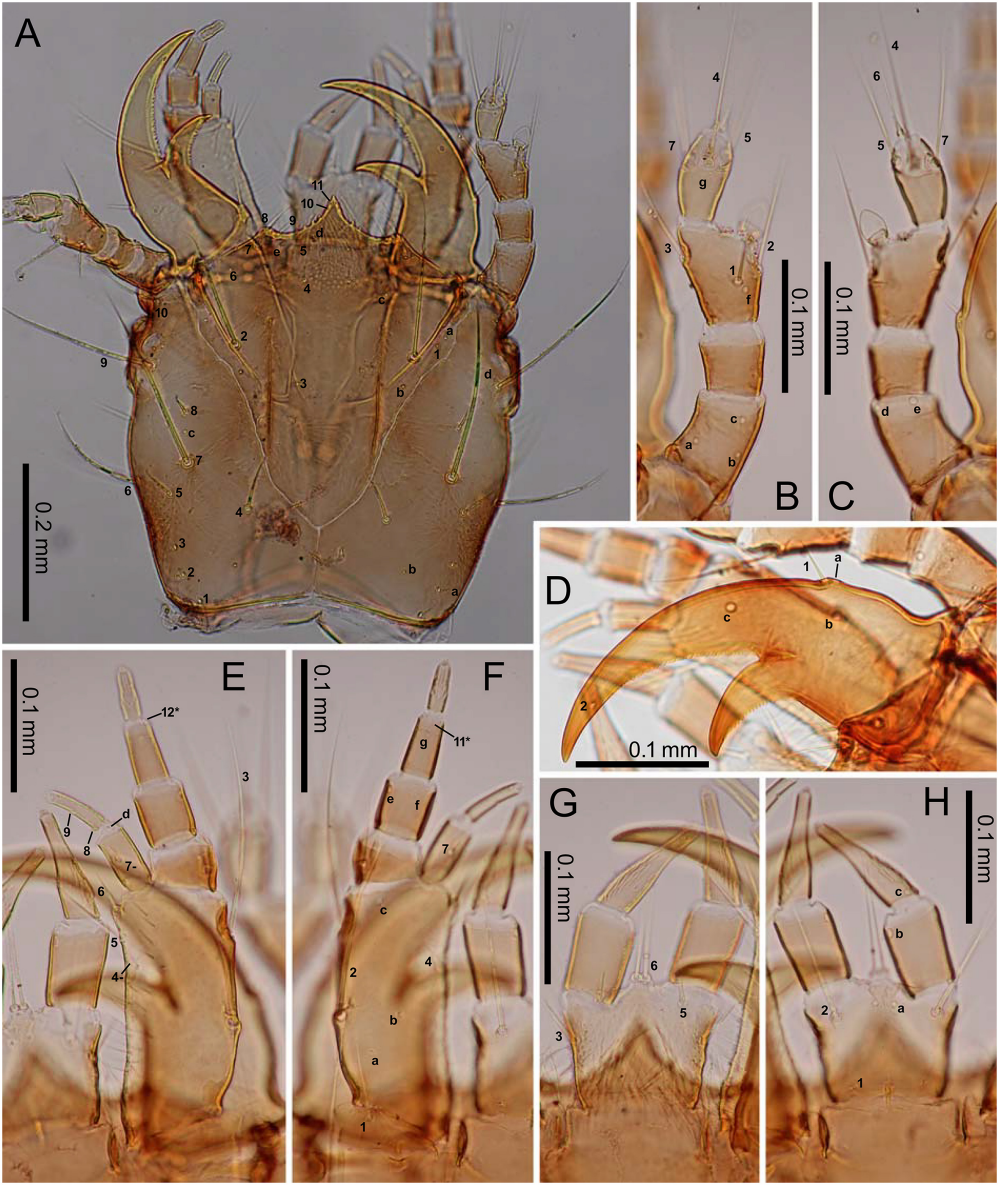
First-instar *E*. *sugai*. (A) Head capsule (dorsal view), (B) antenna (dorsal view), (C) antenna (ventral view), (D) right mandible (dorsal view), (E) maxilla (dorsal view), (F) maxilla (ventral view), (G) labium (dorsal view), (H) labium (ventral view). The homology of characters marked with an asterisk (*) is uncertain, and in Fig 3E, characters with a hyphen (-) are on the ventral side.

**Fig 4:**
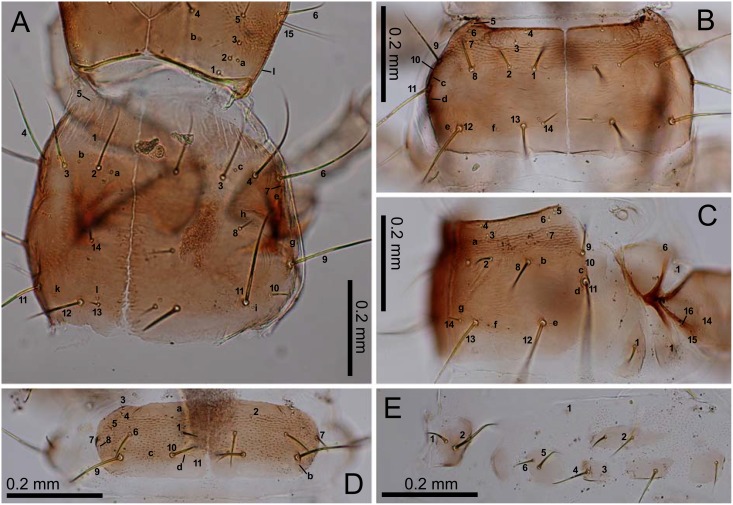
First-instar *E*. *sugai*. (A) Head capsule (part) and pronotum (right dorsolateral view), (B) mesonotum (dorsal view), (C) mesothorax (right lateral view), (D) abdominal tergite I (dorsal view), (E) median, inner, and outer sternites, and epipleurite.

**Fig 5:**
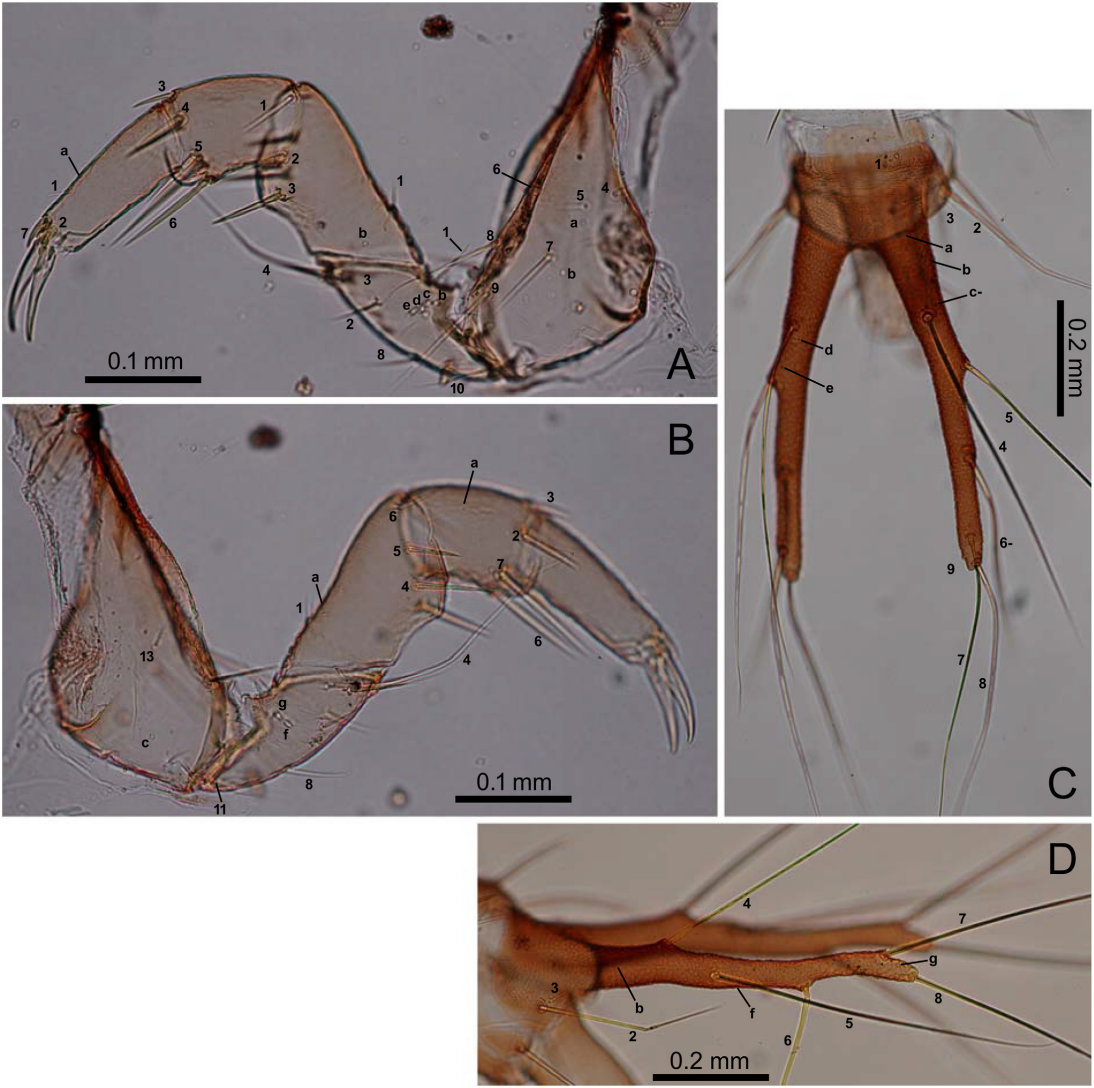
First-instar *E*. *sugai*. (A) Fore leg (anterolateral view), (B) fore leg (posterolateral view), (C) abdominal tergite IX and urogomphi (dorsal view), (D) abdominal tergite IX and urogomphi (left lateral view). In Fig 5C, characters with a hyphen (-) are on the dorsal side.

**Fig 6:**
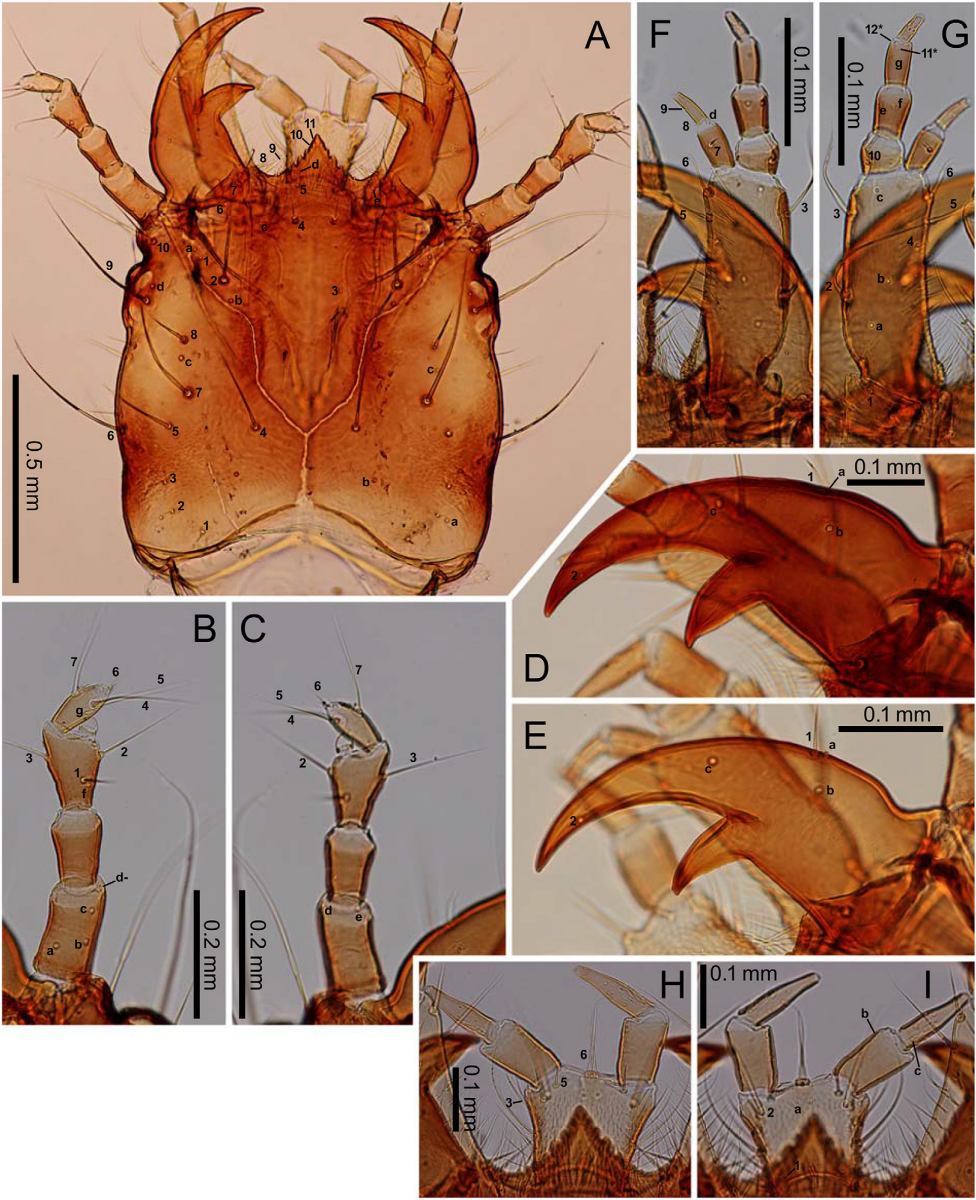
Second- and third-instar *E*. *sugai*. (A) Head capsule of third-instar (dorsal view), (B) antenna of third-instar (dorsal view), (C) antenna of third-instar (ventral view), (D) right mandible of third-instar (dorsal view), (E) right mandible of second-instar (dorsal view), (F) maxilla of third-instar (dorsal view), (G) maxilla of third-instar (ventral view), (H) labium of third-instar (dorsal view), (I) labium of third-instar (ventral view). The homology of characters marked with an asterisk (*) is uncertain, and in Fig 6B, the characters with a hyphen (-) is on the ventral side.

**Fig 7:**
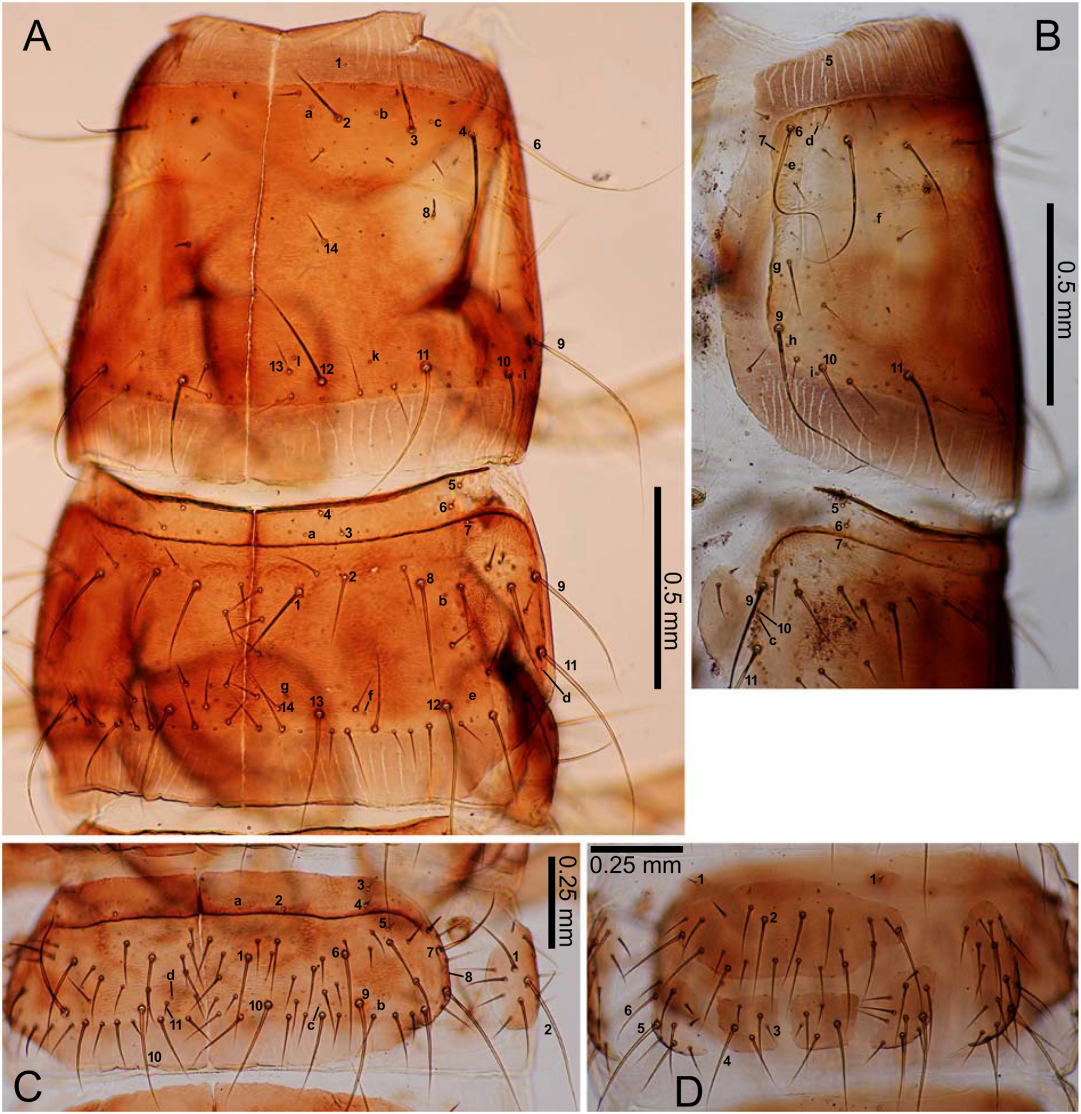
Third-instar *E*. *sugai*. (A) Pro- and mesonotum (right dorsolateral view), (B) Pro- and mesonotum (part) (left lateral view), (C) abdominal tergite I and epipleurite (right dorsolateral view), (D) median, inner, and outer sternites.

**Fig 8:**
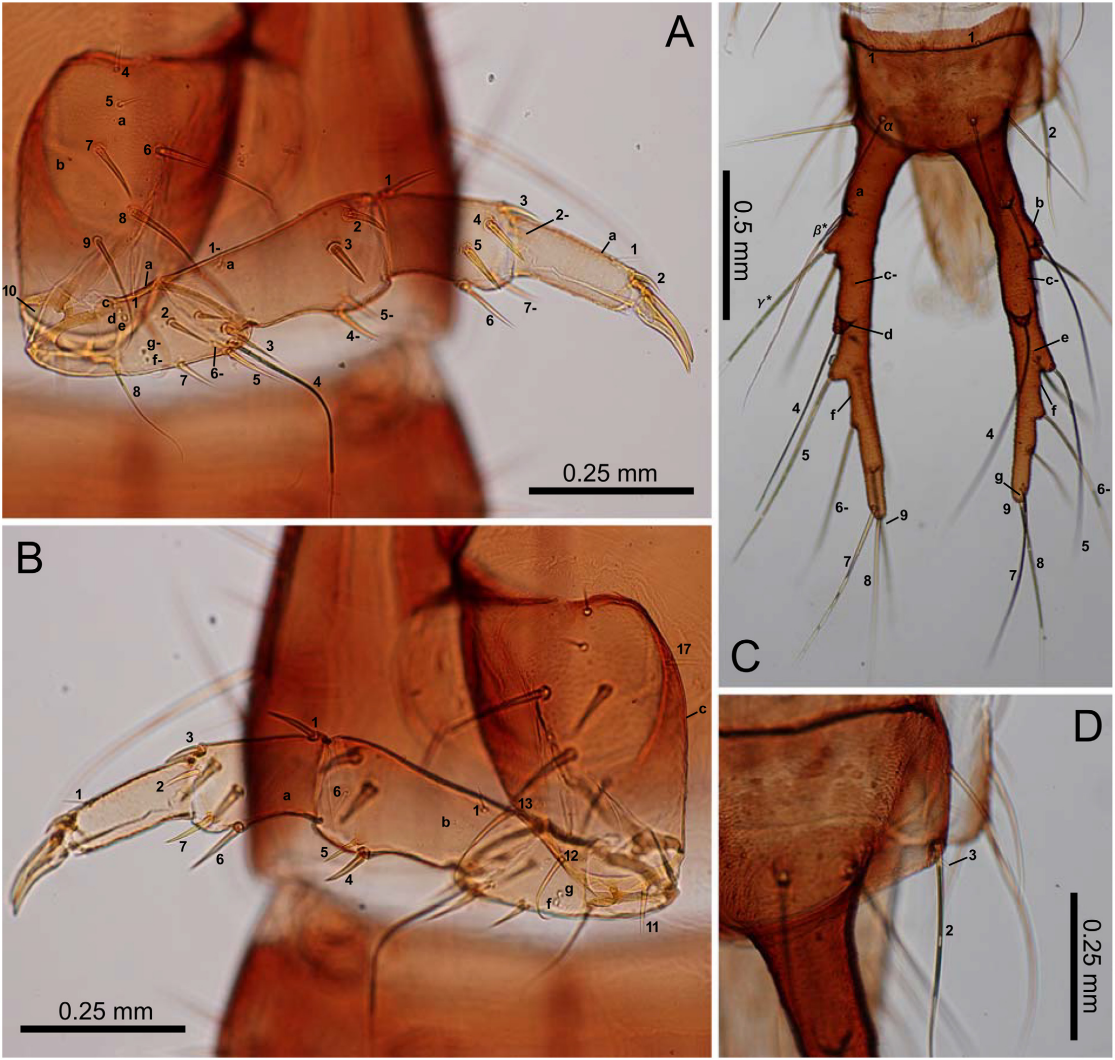
Third-instar *E*. *sugai*. (A) Fore leg (anterolateral view), (B) fore leg (posterolateral view), (C) abdominal tergite IX and urogomphi (dorsal view), (D) abdominal tergite IX and urogomphi (part, dorsal view). The homology of characters marked with an asterisk (*) is uncertain, and characters with a hyphen (-) are on the posterolateral side in Fig 8A and on the ventral side in Fig 8C.

#### Eggs

Immediately after laying (<24 h), 0.5–0.6×1.1–1.2 mm in diameter and light cream in color; just before hatching, 0.65×1.3 mm in diameter and, overall, yellowish-cream in color with the following characters of the embryo visible through the shell: the egg-bursters and their surrounding regions grey in color, brown stemmata, and yellowish-brown mandibular and retinacular apices.

#### Larvae

*Characters present in all instars*: head capsule with a dark-brown dorsal surface, except for the lateral areas behind the stemmata that are roughly surrounded by PA_6_–PA_9_, and the posterior area near the base, the anterior margin of which lies posterior to PA_b_; dark-brown antennae and mandibles; yellowish-brown maxilla, labium, other parts of the head capsule, and the legs; blackish-gray sclerites on the meso- and meta-thorax and abdomen; dark-gray urogomphi; grayish white membranous parts (Figs 4–6). Most primary setae and pores present, but at least LA_5_ and PR_j_ absent. Secondary setae and pores present on antennomere II (one pore on the ventral side), maxillary palpomere IV (5–7 minute setae/pores), galeomere II (one seta on the middle of the inner side, and one pore on the outer side near the apex). Head capsule subquadrate, the length along the median line as long as the width at the widest point. The frontale V-shaped at the base, with the posterior end at the level of the (approximately) basal one-fifth of the head capsule (along the median line of that capsule); the nasale triangular, distinctly prominent; the length from the tip along the median line to the level of FR_5_ as long as that along the median line from the level of FR_5_ to FR_4_; 2–4 additional teeth on the lateral margins posterior to FR_10_; the adnasale sloping at less than 45°. The parietale with six stemmata; the lateral side (viewed dorsally) swollen at the levels of PA_10_ and the stemmata, slightly sinuate behind PA_6_; the cervical groove absent; a coronal suture present. Antennae as long as the mandible; antennomeres I and III subequal in length, longer than antennomeres II and IV; antennomeres I and II cylindrical; antennomere IV and the sensorial appendage on antennomere III claviform. The mandible moderately arcuate anteriorly, the basal width 0.35–0.4-fold the length of the mandible along the median line; the retinaculum located slightly behind the middle of the mandible, with the basal width one-third that of the mandible and the length along the median line 20–25% that of the mandible. The maxilla with a stipe longer than palpomeres I–IV combined; palpomeres II and III and galeomere I subequal in length, longer than palpomeres I and IV; membranous notch absent; lacina absent; gMX with at least 20 setae. Labium with a subquadrate prementum; prementum slightly wider anteriorly; palpomere II slightly longer than palpomere I, but shorter than the prementum; the ligula small; palpomere II with >10 longitudinal channels on the outer side of the basal 75%; the length of the channels approximately half the diameter of the basal end of palpomere II. The thoracic nota and the abdominal tergites transverse; the anterior border of the mesonotum with a distinct edge; the pronotum without distinct notal carina. The urogomphi fused to tergite IX, slightly thinner posteriorly, longer than the pygidium and the head capsule. All legs with two slightly arcuate simple claws subequal in length.

*Characters restricted to first-instars*: Head width 0.47–0.51 mm (mean±SD: 0.49±0.01 mm, n = 10). The urogomphi 0.58–0.67 mm (mean±SD: 0.64±0.03 mm, n = 11). Pronotum entirely blackish gray. Head capsule widest at the stemmata; the lateral sides moderately arcuate posteriorly, with the width at the level of PA_2_ narrower than that at the level of PA_10_; the posterior margin slightly arcuate anteriorly. The frontale with longitudinal keel-like egg-bursters; additional teeth on the nasale widely spaced, with the interval between teeth as wide as the width of the teeth; the adnasale slightly sloping. Antennomere IV longer than antennomere II. Terebra with minute teeth at the basal 2/3; the posterior edge of retinaculum arcuate, with minute teeth at the basal 4/5. Maxillary palpomeres I and IV subequal in length; galeomere I slightly shorter than galeomere II; gMX with 20–35 setae. Notal and abdominal tergal carinae absent.

*Characters restricted to older instars*: Head width 0.59–0.63 mm (mean±SD: 0.62±0.02 mm, n = 4) in second instars, 0.83–0.89 mm (mean±SD: 0.86±0.03 mm, n = 4) in third instars. Urogomphi 0.80–0.93 mm (mean±SD: 0.89±0.06 mm, n = 4) in second instars, 1.16–1.28 mm (mean±SD: 1.23±0.05 mm, n = 4) in third instars. Additional secondary setae present on the parietale (seven on the ventral side, between PA_11_ and PA_i_, between PA_12_ and PA_17_, anteromedial to PA_j_, behind PA_n_, medial or anteromedial to PA_k_, posterolateral to PA_k_, and posteromedial to PA_15_), the pronotum (numerous in the discal area, mainly around the primary setae and pores), the meso- and meta-notum (very numerous in the discal area, mainly around the primary setae and pores), abdominal tergites I–VIII (numerous on I–VIII in L2; very numerous on I–VII and numerous on VIII in L3; covering the entire surfaces of the discal area), abdominal tergite IX and the urogomphi (one, occasionally two, on the lateral margin in front of UR_2_, one on the lateral side at the base of urogomphi, UR_α_, UR_β_, UR_γ_, one on the ventrolateral side behind UR_γ_, one on the ventral side at the level of UR_e_, and one on the ventral side between UR_6_ and UR_7_), the pygidium (few on the dorsal, numerous on the ventral side), the episternum (one), the prosternite (4–7 near PS_1_), the trochantin (one), the pleurite (2–4), the median sternites (1–2 on I and 12–14 on II–VII in L2, few on I and 20–24 on II–VII in L3), the sternal sclerites (numerous, occasionally relatively numerous on segment VIII, few on segment IX), the inner sternite (1–2 in L2, 2–3 in L3), the outer sternite (2–5 in L2, 5–9 in L3), the hypopleurite (4 on segment I, 8–11 on II–VII, 5–7 on VII in L2; 8 on I, 15–23 on II–VII, and 13–14 on VIII in L3), the epipleurite (4–10 on II–VIII and 1–3 on IX in L2, 13–21 on II–VIII and 5–7 in L3), the trochanter (one between TR_2_ and TR_3_). Pronotum large yellowish-brown area on both lateral sides; other parts of pronotum blackish-gray. Head capsule widest at the level between PA_6_ and PA_7_, with the lateral sides subparallel; the width at the level of PA_2_ wider than that at the level of PA_10_; the posterior margin moderately (second-instar) or strongly (third-instar) arcuate anteriorly. Additional teeth on the nasale closely spaced, with virtually no interval; the adnasale moderately sloping. Antennomere IV shorter than antennomere II. Terebra almost smooth, occasionally with ambiguous teeth at the basal 1/3; the posterior edge of retinaculum slightly arcuate throughout with ambiguous teeth at the basal 2/3–3/4 (second-instar) or almost straight, bending at the apical 1/6, with smooth surface (third-instar). Galeomere I slightly but distinctly longer than galeomere II; gMX with 20–35 setae in the second instar, >35 setae in the third instar. The pronotum with indistinct notal carina; the notal carinae of the meso- and meta-notum and the tergal carina of the abdomen distinct; the anterior border of the metanotum with a distinct edge but less developed than that of the mesonotum. The urogomphi with the bases of UR_4_, UR_5_, and UR_γ_, and those of the secondary seta behind UR_γ_, markedly swollen.

#### Pupae

Yellowish-white except for compound eyes, which gradually become dark-brown until eclosion. The dorsal side with numerous setae, most of which are absent in the adult: head (four on each lateral side: anterior and posterior supraorbital setae, two additional setae near the posterior supraorbital seta), the pronotum (≈15 on each lateral side: one in the middle, the remaining setae along the outer margin of the pronotum), the meso- and meta-notum (four to five on the middle of each lateral side), the abdominal tergites (four to five on the middle of each lateral side of segments I–V, two on VI–VIII, their lengths as long as those of the two segments combined, their apices somewhat “coiled”), and the epipleurite (two on segments II–VIII).

## Discussion

The results revealed that *E*. *sugai* could lay over 131 eggs. This was the number laid by a female collected in early April, thus early in the reproductive period, and subsequently reared under stable temperature, photoperiod, and oviposition-substrate conditions. Therefore, in the field (under fluctuating conditions), fecundity may be lower. Moreover, as reported for other carabids, fecundity may differ among individuals. Indeed, the egg-laying rate per day of the 2014 female was 1.8-fold higher than that of the 2013 female. Although the other 2014 female laid only four eggs, this was likely an artifact caused by unidentified physiological/environmental conditions and does not reflect the actual fecundity. Compared with other carabids, the fecundity of *E*. *sugai* is not markedly low (Table 4). This suggests that low fecundity is not the principal reason why *E*. *sugai* is endangered.

**Table 4:** Fecundity of various taxa of Carabidae.

Taxa	Total number of eggs laid in a single breeding season	Egg-laying rate (eggs/day)	Source
*Cychrus schmidti* Chaudoir	20 (mean from four females)	0.33	[14]
*Carabus asperatus* (Dejean)	241 (from a female)	4.46 (from a female)	[12]
*Ca*. *auratus* Linnaeus	56 (mean)	–	[15]
*Ca*. *cancellatus* Illiger	45 (mean)	–	[15]
*Ca*. *granulatus* Linnaeus	41 (mean)	–	[15]
*Ca*. *stenocephalus stenocephalus* Lucas	288 (from a female)	5.88 (from a female)	[12]
*Ca*. *stenocephalus susicus* Antoine	423–511 (from two females)	4.23–12.46 (from a female)	[12]
*Ca*. *stenocephalus ifniensis* Zarco	342 (from a female)	7.95 (from a female)	[12]
*Ca*. *stenocephalus aliai* Escalera	284 (mean from three females)	3.27 (mean from three females)	[12]
*Ca*. *ulrichi* Germar	22 (mean)	–	[15]
*Abax ovalis* (Duftschmid)	≈15	–	[15]
*Ab*. *parallelus* Duftschmid	32–48 (mean)^1^		[15]
*Poecilus chalcites* (Say)	351 (mean)	–	[15] as *Pterostichus chalcites* Say
*Po*. *cupreus* (Linnaeus)	–	≈0.2–0.9^2^	[16]
*Po*. *fortipes* (Chaudoir)	54 (from a pair)	–	[17]
*Po*. *versicolor* (Sturm)	77.5 (mean in the first year)	–	[18]
*Pterostichus angustatus* (Duftschmid)	105 (mean of bred females); 136 (mean of trapped females); 320 (max. of bred females); 261 (max. of trapped females)	–	[15]
*Pt*. *oblongopunctatus* (Fabricius)	48 (mean of bred females); 65 (mean of trapped females); 129 (max. of bred females); 105 (max. of trapped females)	–	[15]
*Amara macronota* (Solsky)	–	1.56 (mean in a pure-animal diet); 1.18 (mean in a mixed-seed diet); 3.02 (mean in a mixed animal/seed diet)	[19]
*Am*. *similata* (Gyllenhal)	–	0.94 (mean in a pure-animal diet); 3.12 (mean in a pure-seed diet); 3.36 (mean in a mixed animal/seed diet)	[20]
*Anisodactylus punctatipennis* Morawitz	–	1.25 (mean in a pure-animal diet); 4.68 (mean in a mixed-seed diet); 5.87 (mean in a mixed animal-seed diet)	[21]
*Harpalus rufipes* (Degeer)	–	0.020 (mean in a pure-animal diet); 0.502 (mean in a pure-seed diet); 0.650 (mean in a mixed animal/seed diet)	[22]
*Agonum sexpunctatum* (Linnaeus)	27.6 (mean in the first year)	–	[15] as *Agonum assimile* (Motschulsky)

^1^Calculated from the information that “an average of 16 per batch” and “females lay twice, at intervals of one to two months, and occasionally even three times”.

^2^Calculated from the values in Fig 2.

Although some eggs were laid on moistened moss and mud, this is probably abnormal or uncommon. This is because the eggshell is as soft as those of other carabids that lay eggs in the soil, and because all other eggs were observed to be laid in mud. It might be assumed that eggs laid in mud would suffocate. However, ovipositioning into mud has been reported in other riparian carabids (*Carabus clatratus*: [23]; *Bembidion velox*: [24]; *Nebria yatsugatakensis*: [25]). Notably, in *B*. *velox*, it has been shown experimentally that eggs survive better under flood conditions [24]. This may also be true of *E*. *sugai*. If so, maintenance of a suitable ovipositioning environment is vital in terms of conservation. Experimental studies exploring ovipositioning substrate preferences and the relationships between flooding and offspring survival are required; such work has been performed in other riparian carabids [23,24,26].

Larval rearing showed that *E*. *sugai* larvae are insect larvae feeders. As diet types/conditions differ between the laboratory and the field, this result requires further investigation. In the laboratory, mealworms cut into pieces were provided to *E*. *sugai* larvae. In the field, however, much smaller live animals will be consumed. The observed high mortality of first-instar larvae (Tables 2 and 3) may be an artifact due to this difference in diet type/conditions between the laboratory and the field, because mealworms cut into pieces become moldy more readily than do live prey. Examinations of candidate prey in the *E*. *sugai* habitat and subsequent laboratory rearing experiments would elucidate this issue. Moreover, stable isotope analysis of field-collected samples would be helpful, because the field diets of other carabids have been explored using this method [27,28]. This study describes the preimaginal morphology of *E*. *sugai*, allowing field samples to be identified and collected. The earthworm diet was toxic to larvae. Although some carabids eat a non-optimal diet as a supplemental diet and gain some benefits (e.g., a pure-animal diet for granivorous carabids [29]), *E*. *sugai* larvae will not eat earthworms even as a supplemental diet in the field.

The larval morphology of *E*. *sugai* is similar to that of consubgeneric species described previously [30]. However, the morphology differs from that of the sympatric *E*. *punctatus* (belonging to another subgenus) in terms of the shape of the mandible, which is less arcuate in *E*. *sugai* than *E*. *punctatus* [for mandible shape of *E*. *punctatus*, [13] Fig 1A (dorsal view of right mandible of the first-instar) and [31] (coordinates for the contour of right mandible of all instars)]. For eggs and pupae, to my knowledge, this is the first morphological description for a species of the subfamily Elaphrinae. In terms of the pupae, compared with other carabids (*Cychrus schmidti* [14]; *Carabus asperatus*, *Ca*. *stenocephalus susicus*, *Ca*. *stenocephalus aliai* [12]; *Ca*. *rugosus baeticus* [32]; *Ca*. *blaptoides* [18]; *Ca*. *vanvolxemi* [33]; *Loricera pilicornis* [34]; *Loxandrus oophagus* [35]; *Macracanthus brevicillus* [36]; *Galerita janus* [37]; *Agonum quandripunctatum* [38]), the somewhat coiled apices of the setae on the abdominal tergites are unique to *E*. *sugai*. The relative length of the setae (compared with the length of the abdominal segments) is also unusual. The length is twice that of an abdominal segment. In all but one other species, the setal length is shorter than that of an abdominal segment. The exception is *Loricera pilicornis* [34], in which the setae are twice as long as an abdominal segment, similar to *E*. *sugai*. As both *E*. *sugai* and *L*. *pilicornis* are species of saturated soil habitats, long setae on the abdominal tergites may be associated with the habitat conditions.

Most *Elaphrus* species occur in marsh/riparian environments, which are often damaged by human activity and are thus endangered. Among such species, *E*. *viridis* Horn of North America is one of the four carabids included in the IUCN Red List and is classified as “Critically Endangered” [39]. Therefore, *E*. *viridis* has been regarded as the most endangered *Elaphrus* worldwide. However, given its current distribution and the minimal conservation efforts being made, *E*. *sugai* is probably more endangered. According to available information, *E*. *viridis* is thought to occupy a maximal area of 28 km^2^ [40], which is nearly identical to the area of the Watarase wetland (28.5 km^2^), within which only a few small sites serve as appropriate *E*. *sugai* habitats (note that a large artificial lake was excluded when calculating the area of the Watarase wetland). Thus, the area of suitable habitat is apparently smaller in the case of *E*. *sugai* than in that of *E*. *viridis*. More importantly, conservation efforts differ markedly between the two species. *E*. *viridis* is classified as “threatened” by the U.S. Fish and Wildlife Service; specimen collection and habitat disturbance are strictly prohibited. For *E*. *sugai*, however, collection is not prohibited, and no law seeks to protect *E*. *sugai* habitats. As noted in the Introduction, the *E*. *sugai* situation is deteriorating rapidly, and conservation measures are urgently needed. The current data on reproductive ecology and preimaginal morphologies constitute valuable basic information facilitating conservation.
